# *Fomitiporellacrystallina* sp. nov. (Basidiomycota, Hymenochaetales) from China

**DOI:** 10.3897/BDJ.11.e95945

**Published:** 2023-02-09

**Authors:** Xiao-Hong Ji, Longfei Fan

**Affiliations:** 1 College of Pharmacy and Life Sciences, Jiujiang University, Jiujiang, China College of Pharmacy and Life Sciences, Jiujiang University Jiujiang China; 2 College of Plant Protection, Gansu Agricultural University, Lanzhou, China College of Plant Protection, Gansu Agricultural University Lanzhou China

**Keywords:** Taxonomy, phylogeny, wood-decaying fungi

## Abstract

**Background:**

*Fomitiporella* is an important genus of wood-decaying fungi. Many new species were revealed in the last five years, based on morphological characters and molecular data. During a study on the taxonomy of *Fomitiporella*, two specimens from China were investigated, which have morphological characteristics close to *Fomitiporella*. After morphological examinations and phylogenetic analyses, a new species was confirmed to be a member of the *Fomitiporella* clade.

**New information:**

*Fomitiporellacrystallina* sp. nov. is described and illustrated as a new species, based on morphological characters and molecular evidence. It has perennial, irregular, pileate basidiocarps, an indistinct subiculum (ultrathin to almost lacking), lack of any kind of setae, has brownish, thick-walled basidiospores and causes a white rot. A molecular study, based on the combined ITS (internal transcribed spacer region) and nrLSU (the large nuclear ribosomal RNA subunit) dataset, supports the new species in *Fomitiporella*. The differences between the new species and phylogenetically related and morphologically similar species are discussed. A key to species with pileate to effused-reflexed basidiocarps of *Fomitiporella* is given.

## Introduction

*Fomitiporella* Murrill (1907), typified by *F.umbrinella* Murrill, was proposed to accommodate polypores with perennial, resupinate and adnate basidiocarps, a thin subiculum, stratified tubes and subglobose and brown basidiospores. However, it was not treated as an independent genus, but as a synonym of *Phellinus* Quél. by most mycologists (Ryvarden and Johansen 1980, Larsen and Cobb-Poulle 1990, Ryvarden 1991, Ryvarden and Gilbertson 1994, Dai 1999, Núñez and Ryvarden 2000) until Wagner and Fischer (2002) confirmed *Fomitiporella* as a distinct genus in Hymenochaetaceae, based on nrLSU DNA sequence data. *Fomitiporella* was accepted as a monophyletic genus by molecular phylogeny (Zhou 2014). Ji et al. (2017) broadened its concept to accommodate species with resupinate to effused reflexed and annual basidiocarps. Recently, *Arambarria* Rajchenb. & Pildain, *Phellinotus* Drechsler-Santos, Robledo & Rajchenb. and *Rajchenbergia* Salvador-Montoya were established and formed three clades in phylogeny of *Fomitiporella* (Rajchenberg et al. 2015, Drechsler-Santos et al. 2016, Salvador-Montoya et al. 2020). However, based on molecular data and morphological re-interpretation, these genera were treated as synonyms of *Fomitiporella* (Wu et al. 2022). Furthermore, several species with pileate basidiocarps were introduced into *Fomitiporella* (Wu et al. 2022).

During a study on the taxonomy of *Fomitiporella*, two collections from China were investigated. After morphological examinations and phylogenetic analyses, a new species was confirmed to be member of the *Fomitiporella* clade. In this paper, we describe and illustrate the new species. In addition, an identification key to the worldwide species with pileate to effused-reflexed basidiocarps of *Fomitiporella* is provided.

## Materials and methods

### Morphology

Studied specimens are deposited in the herbaria of Southwest Forestry University (SWFC) and the Mycological Herbarium of Jiujiang University (MHJU). Microscopic examination follows Dai (2010) and colour terms follow Petersen (1996). Microscopic structures were photographed using a Nikon Digital Sight DS-Fi1 camera. Microscopic measurements were made from slide preparations stained with Cotton Blue and Melzer’s reagent. In the text, the following abbreviations were used: KOH = 5% potassium hydroxide, IKI = Melzer’s reagent, IKI– = neither amyloid nor dextrinoid, CB = Cotton Blue, CB(+) = cyanophilic after 12 hours stained with Cotton Blue, CB– = acyanophilic, L = mean spore length, W = mean spore width, Q = variation in the ratios of L/W between specimens studied, n = number of spores measured from given number of specimens.

### Molecular phylogeny

**DNA extraction, amplification and sequencing**

A CTAB rapid plant genome extraction kit (Aidlab Biotechnologies Co., Ltd., Beijing, China) was used to extract total genomic DNA from dried specimens following the manufacturer’s instructions with some modifications (Chen et al. 2016). The DNA was amplified with the primers: ITS4 and ITS5 for ITS (White et al. 1990) and LR0R and LR7 for nrLSU. The PCR protocol for ITS was as follows: initial denaturation at 95°C for 3 min, followed by 35 cycles at 94°C for 40 s, 54°C for 45 s and 72°C for 1 min and a final extension of 72°C for 10 min. The PCR procedure for nrLSU was as follows: initial denaturation at 94°C for 1 min, followed by 35 cycles at 94°C for 30 s, 50°C for 1 min and 72°C for 1.5 min and a final extension of 72°C for 10 min. The PCR products were purified and sequenced at the Changsha Genomics Institute, China, with the same primers. The resulted ITS and nrLSU sequences were submitted to GenBank (http://www.ncbi.nlm.nih.gov/genbank).

**Phylogenetic analysis**

Besides the newly-generated sequences, additional sequences from a previous phylogenetic study on *Fomitiporella* (Wu et al. 2022) were included in the current dataset (Table 1). The dataset, outgrouped by *Inocutisdryophila* (Berk.) Fiasson & Niemelä (Wu et al. 2022), was aligned using BioEdit (Hall 1999) and ClustalX 1.83 (Chenna et al. 2003). Alignment was manually adjusted to allow maximum alignment and to minimise gaps. Maximum Likelihood (ML) and Bayesian Inference (BI) were employed to perform phylogenetic analysis of the aligned dataset.

The two phylogenetic analysis algorithms generated nearly congruent topologies for the dataset and, thus, only the topology from the ML analysis is presented along with statistical values from the ML and BI algorithms (BS not less than 50% and BPP not less than 0.9) at the nodes. The tree was visualised in TreeView 1.6.6 (Page 1996).

## Taxon treatments

### Fomitiporella crystallina

X.H. Ji sp. nov.

85AE1DC1-A55F-5F01-B8EB-9789D117A697

844077

#### Materials

- **Type status:** Holotype. **Occurrence:** recordNumber: CL Zhao 9453; recordedBy: Zhao Chang-Lin; occurrenceID: 8A2E5C2A-F223-5678-9985-3E269FEF13D5; **Taxon:** scientificName: *Fomitiporellacrystallina*; acceptedNameUsage: *Fomitiporellacrystallina* X.H. Ji, 2022, sp. nov.; parentNameUsage: *Fomitiporella*, Murrill, 1907; kingdom: Fungi; phylum: Basidiomycota; class: Agaricomycetes; order: Hymenochaetales; family: Hymenochaetaceae; genus: Fomitiporella; specificEpithet: Crystallina; taxonRank: species; verbatimTaxonRank: species; scientificNameAuthorship: X.H. Ji; **Location:** continent: Asia; country: China; stateProvince: Yunnan Province; municipality: Puer; locality: Huilianghe,Puer,Yunnan Province,China; verbatimElevation: 1400 m; locationRemarks: label transliteration: "Yunnan, Jingdong Yi Autonomous County, Huilianghe, 2019 1.5, Zhao Changlin et al."; [云南会良河村 2019.01.05, 赵长林等]; georeferenceProtocol: label; **Identification:** identifiedBy: Ji Xiaohong; dateIdentified: 2019; **Event:** samplingProtocol: collecting; eventDate: 2019.1.5; **Record Level:** language: en; institutionCode: the herbaria of Southwest Forestry University (SWFC) and the Mycological Herbarium of Jiujiang University (MHJU); collectionCode: Fungi; ownerInstitutionCode: the herbaria of Southwest Forestry University (SWFC) and the Mycological Herbarium of Jiujiang University (MHJU)
- **Type status:** Isotype. **Occurrence:** recordNumber: CL Zhao 9567; recordedBy: Zhao Chang-Lin; occurrenceID: BDA3DEBA-4C54-545A-AF79-7FB591AD7C9E; **Taxon:** scientificName: *Fomitiporellacrystallina*; acceptedNameUsage: *Fomitiporellacrystallina* X.H. Ji, 2022, sp. nov.; parentNameUsage: *Fomitiporella* Murrill, 1907; kingdom: Fungi; phylum: Basidiomycota; class: Agaricomycetes; order: Hymenochaetales; family: Hymenochaetaceae; genus: Fomitiporella; specificEpithet: Crystallina; taxonRank: species; verbatimTaxonRank: species; scientificNameAuthorship: X.H. Ji; **Location:** continent: Asia; country: China; stateProvince: Yunnan Province; municipality: Puer; locality: Huilianghe,Puer,Yunnan Province,China; verbatimElevation: 1400 m; locationRemarks: label transliteration: "Yunnan, Jingdong Yi Autonomous County, Huilianghe, 2019 1.5, Zhao Changlin et al."; [云南会良河村 2019.01.05, 赵长林等]; georeferenceProtocol: label; **Identification:** identifiedBy: Ji Xiaohong; dateIdentified: 2019; **Event:** samplingProtocol: collecting; eventDate: 2019.1.5; **Record Level:** language: en; institutionCode: the herbaria of Southwest Forestry University (SWFC) and the Mycological Herbarium of Jiujiang University (MHJU); collectionCode: Fungi; ownerInstitutionCode: the herbaria of Southwest Forestry University (SWFC) and the Mycological Herbarium of Jiujiang University (MHJU)

#### Description

Fruiting body (Fig. 2): Basidiocarps perennial, pileate to pendent, hard corky and without odour or taste when fresh, woody hard and medium in weight when dry; pilei irregular, projecting up to 8 cm, 4 cm wide and 3.5 cm thick at base; pileal surface black to dark brown at the margin, narrowly concentrically sulcate, glabrous, with a very hard black crust 1–5 mm thick; margin obtuse. Pore surface greyish-brown when fresh, becoming deep olive when dry; sterile margin yellowish-brown, up to 2 mm wide; pores circular to angular, 5–8 per mm; dissepiments thin, more or less entire to slightly lacerate; tubes woody hard, concolorous with pores, each layer up to 2 mm deep, white mycelial strands present in old tubes. Subiculum very thin to almost lacking.

Hyphal structure: Hyphal system dimitic; generative hyphae simple septate; skeletal hyphae dominant; tissue darkening but otherwise unchanged in KOH.

Tubes: Generative hyphae frequent, hyaline to pale yellow, thin- to slightly thick-walled, occasionally branched, frequently simple septate 1.5–2.5 μm in diam; skeletal hyphae golden yellow to pale brown, thick-walled with a wide to narrow lumen, unbranched, aseptate, interwoven, 2–3.5 μm in diam; setae and cystidioles absent; hymenium collapsed in the studied material, basidia and basidioles not seen; small or large rhomboid crystals abundant.

Spores: Basidiospores broadly ellipsoid, golden yellow, thick-walled, smooth, IKI–, CB(+), (4.1–)4.3–4.7(–4.9) × (3–)3.2–3.9(–4) µm, L = 4.50 µm, W = 3.42 µm, Q = 1.20–1.35 (n = 60/2) (Fig. 3).

#### Etymology

*Crystallina*, referring to the species having abundant crystals.

## Identification Keys

### An identification key to the accepted species with pileate to effused-reflexed basidiocarps of *Fomitiporella*.

**Table:** 

1	Basidiocarps pileate	2
–	Basidiocarps effused-reflexed	6
2	Basidiospores 4–5 µm long	3
–	Basidiospores 5–7.5 µm long	4
3	Pores 8–9 per mm	*F.queenslandica*
–	Pores 5–8 per mm	***F.crystallina***
4	Context with a granular core, black line absent	*F.neoarida* (Drechsler-Santos & Robledo) Y.C. Dai & F. Wu
–	Context without a granular core, black line present	5
5	Baisiospores 5‒5.5 × 3.5‒4.5 μm	*F.piptadeniae* (Teixeira) Y.C. Dai & F. Wu
–	Baisiospores 7–7.5 × 5.5–6 μm	*F.badius* (Cooke) Y.C. Dai & F. Wu
6	Basidiocarps annual to biennial	7
–	Basidiocarps perennial	8
7	Context homogeneous; hyphal system monomitic; Neotropical species	*F.destruens* (Rajchenb. & Pildain) Y.C. Dai
–	Context duplex; hyphal system dimitic; Asian species	*F.chinensis* (Pilát) Y.C. Dai, X.H. Ji & Vlasák
8	Pores 1–4 per mm	9
–	Pores 4–9 per mm	10
9	Basidiospores 5.2–6.5 μm long	*F.cyclobalanopsidis* (T.T. Chang & W.N. Chou) Y.C. Dai & F. Wu
–	Basidiospores 7.5–9.5 μm long	*F.shoushana* (T.T. Chang & W.N. Chou) Y.C. Dai & F. Wu
10	Pores 7–9 per mm; basidiospores 3–4 µm long	*F.caryophylli* (Racib.) T. Wagner & M. Fisch.
–	Pores 4–7 per mm; basidiospores 4–4.8 µm long	*F.vietnamensis* Y.C. Dai, X.H. Ji & J. Vlasák

## Analysis

**Figure 1.**
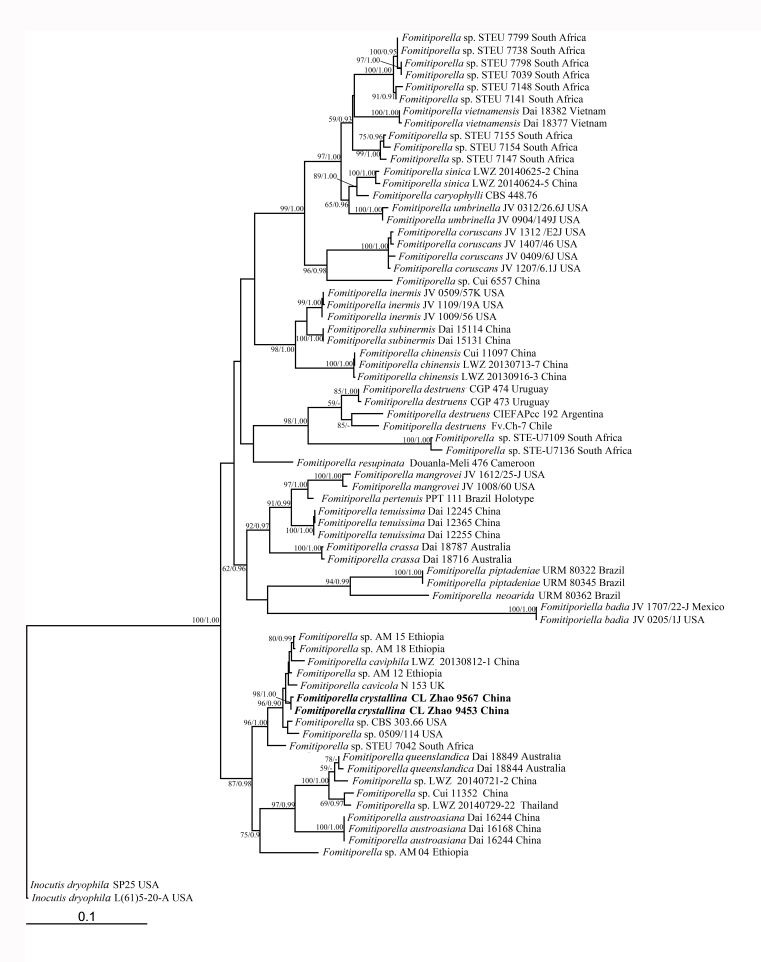
Phylogeny of *Fomitiporella* inferred from the ITS and nrLSU dataset. Topology is from ML tree and statistical values (ML/BI) are indicated for each node that simultaneously received BS from ML not below 50%, and BPP from BI not below 0.9.

**Figure 2.**
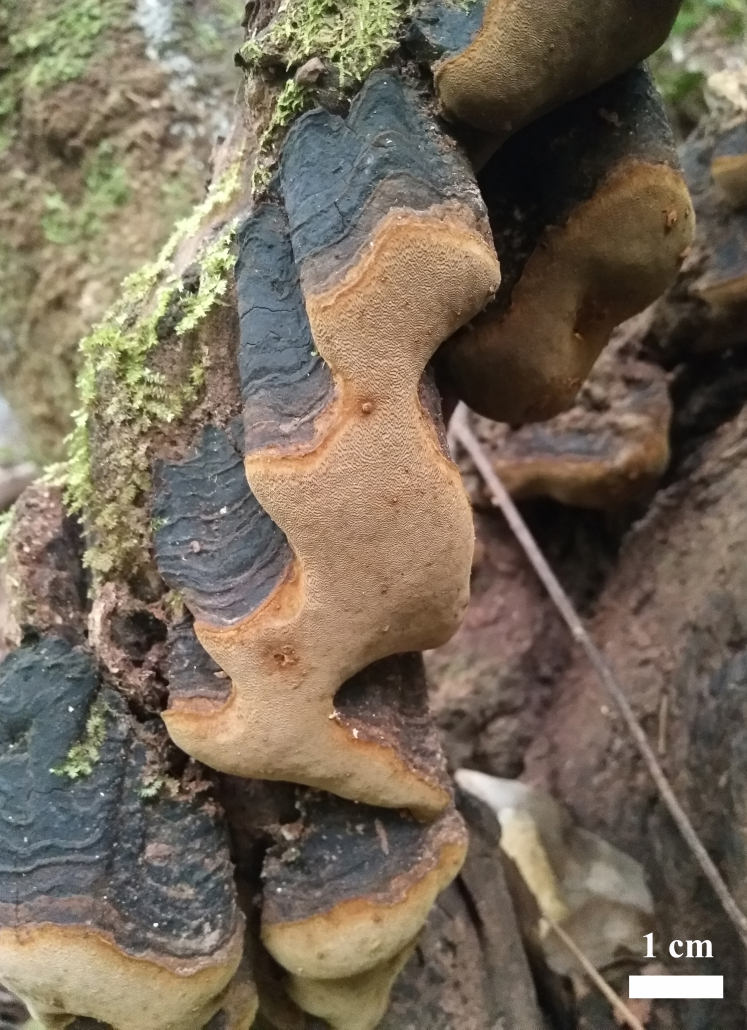
Basidiocarp of *Fomitiporellacrystallina* (CL Zhao 9453). Scalebar: 1 cm.

**Figure 3.**
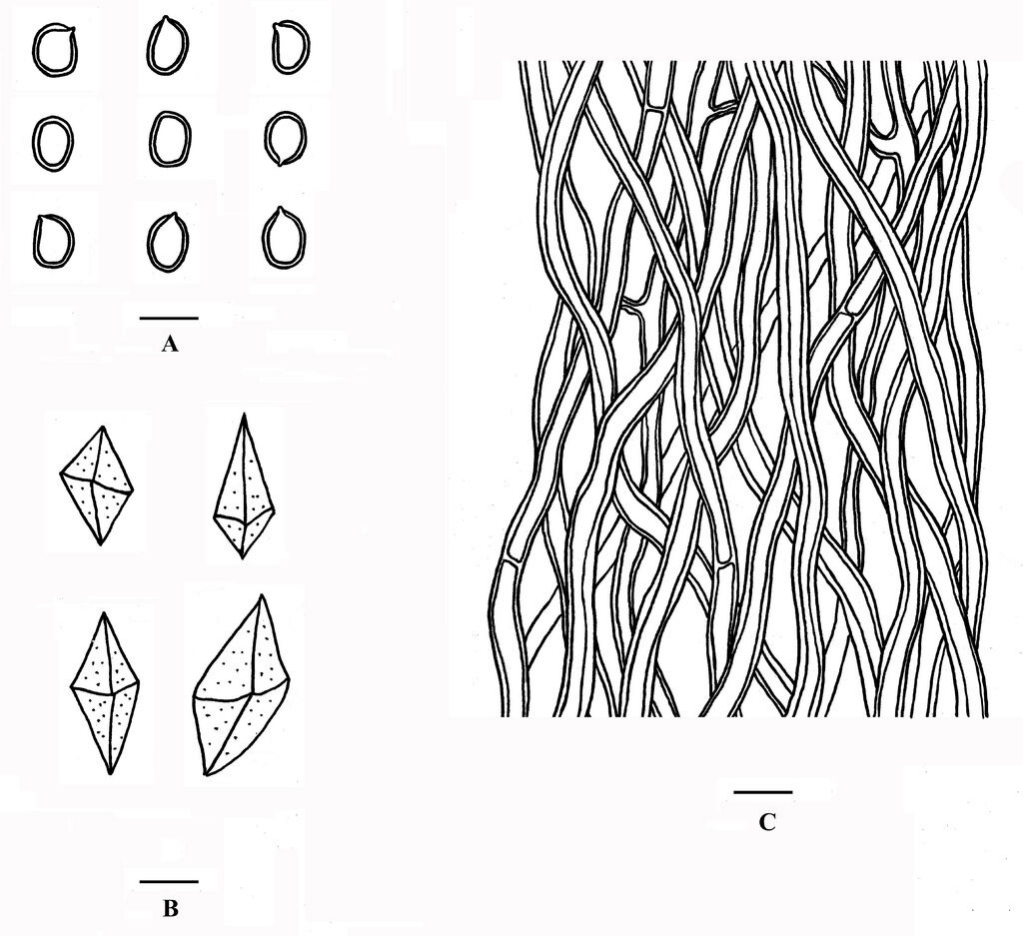
Microscopic structures of *Fomitiporellacrystallina* (CL Zhao 9453). **A** Basidiospores; **B** Rhomboid crystals; **C** Hyphae from trama. Scale bars: 5 μm.

A total of 68 fungal collections, representing 22 species and 22 taxa of *Fomitiporella*, were included in the phylogenetic analyses and two samples of genus *Inocutis* were used as outgroups. The final alignment comprised a total of 1667 base pairs (bp) including 804 of ITS and 863 of nrLSU. Two sampled specimens of the new species, *Fomitiporellacrystallina*, formed a well-supported lineage (BS/PP values 98/1.0), indicating that they are phylogenetically distinct from other species in Fig. 1.

## Discussion

*Fomitiporellacrystallina* has unique morphological characters in *Fomitiporella* and forms a distinct lineage within the *Fomitiporella* clade. Morphologically, *F.crystallina* is similar to *F.queenslandica* Y.C. Dai & F. Wu in sharing perennial, pileate basidiocarps, a dimitic hyphal structure and the same size of basidiospores (4.1–5 × 3–4 μm), whereas the latter has dimidiate pilei and smaller pores (5–8 per mm; Wu et al. (2022)). Moreover, the presence of the cystidioles and a distinct subiculum in *F.queenslandica* makes it different from *F.crystallina*. Phylogenetically, *Fomitiporellacrystallina* is closely related to *F.cavicola* (Kotl. & Pouzar) T. Wagner & M. Fisch. by sharing the perennial basidiocarps, a dimitic hyphal system and the same size range of pores (5–8 per mm), but *F.cavicola* has resupinate basidiocarps and larger basidiospores (4.7–5.5 × 4–4.5 μm). In addition, *F.cavicola* is known from Europe only (Kotlaba and Pouzar 1995).

In conclusion, both morphology and phylogeny support that the specimens, collected from China, are a new species within the genus *Fomitiporella*.

## Supplementary Material

XML Treatment for Fomitiporella crystallina

## Figures and Tables

**Table 1.** Information on the sequences used in this study. Type specimens are shown in bold.

Species	Location	Sample no.	GenBank accession no.	
			ITS	nLSU
*Fomitiporellaaustroasiana*	China	Dai 16244	MG657328	MG657320
*F.austroasiana*	China	Dai 16168	MG657329	MG657321
*F.austroasiana*	Singapore	Dai 17879	MG657330	MG657324
*F.badia*	Mexico	JV 1707/22-J	MW928042	—
*F.badia*	USA	JV 0205/1J	MW928043	—
*F.caryophylli*	India	CBS 448.76	AY558611	AY059021
*F.cavicola*	UK	N 153	—	AY059052
*F.caviphila*	China	LWZ 20130812-1	—	KF729937
*F.chinensis*	China	Cui 11097	KX181310	KX181342
*F.chinensis*	China	LWZ 20130713-7	KJ787817	KJ787808
*F.chinensis*	China	LWZ 20130916-3	KJ787818	KJ787809
*F.coruscans*	USA	JV 1312/E2J	KX181294	KX181333
*F.coruscans*	USA	JV 1407/46	KX181295	KX181332
*F.coruscans*	USA	JV 0409/6J	KX181296	KX181331
*F.coruscans*	USA	JV 1207/6.1J	KX181297	KX181330
*F.crassa*	Australia	Dai 18787	MW924064	MW892735
*F.crassa*	Australia	Dai 18716	MW924063	MW892736
***F.crystallina***	**China**	**CL Zhao 9453**	**ON493552**	**ON493576**
***F.crystallina***	**China**	**CL Zhao 9567**	**ON493553**	ON493577
*F.destruens*	Uruguay	CGP 473	KY907683	KY907687
*F.destruens*	Uruguay	CGP 474	KY907682	KY907692
*F.destruens*	Argentina	CIEFAPcc 192	AY072033	KP347520
*F.destruens*	Chile	Fv.Ch-7	—	DQ459301
*F.inermis*	USA	JV 0509/57K	KX181305	KX181346
*F.inermis*	USA	JV 1109/19A	KX181304	—
*F.inermis*	USA	JV 1009/56	KX181306	KX181347
*F.mangrovei*	USA	JV 1008/60	KX181313	KX181334
*F.mangrovei*	France	JV 1612/25-J	MG657331	MG657325
*F.neoarida*	Brazil	URM 80362	KM211294	KM211286
*F.pertenuis*	Brazil	PPT 111	MG806101	MG806100
*F.piptadeniae*	Brazil	URM 80322	KM211290	KM211282
*F.piptadeniae*	Brazil	URM 80345	KM211291	KM211283
*F.queenslandica*	Australia	Dai 18849	—	MW892737
*F.queenslandica*	Australia	Dai 18844	—	MW892738
*F.resupinata*	Cameroon	Douanla-Meli 476	KJ787822	JF712935
*F.sinica*	China	LWZ 20140625-2	KX181301	KX181320
*F.sinica*	China	LWZ 20140624-5	KX181302	KX181321
*Fomitiporella* sp.	South Africa	STE-U7109	KP279300	—
*Fomitiporella* sp.	South Africa	STE-U7136	JQ038896	—
*Fomitiporella* sp.	China	Cui 6557	KX181303	—
*Fomitiporella* sp.	China	Cui 11352	KX181315	KX181338
*Fomitiporella* sp.	China	LWZ 20140721-2	KX181316	KX181337
*Fomitiporella* sp.	Thailand	LWZ 20140729-22	KX181317	KX181339
*Fomitiporella* sp.	Ethiopia	AM 12	JF895466	JQ910908
*Fomitiporella* sp.	Ethiopia	AM 15	JF895467	JQ910909
*Fomitiporella* sp.	Ethiopia	AM 18	JF895468	JQ910910
*Fomitiporella* sp.	Ethiopia	AM 04	KX181318	KX181335
*Fomitiporella* sp.	South Africa	STEU 7147	JQ038897	—
*Fomitiporella* sp.	South Africa	STEU 7042	JQ038893	—
*Fomitiporella* sp.	South Africa	STEU 7155	KP279297	—
*Fomitiporella* sp.	South Africa	STEU 7154	JQ038894	—
*Fomitiporella* sp.	South Africa	STEU 7148	KP279296	—
*Fomitiporella* sp.	South Africa	STEU 7141	KP279295	—
*Fomitiporella* sp.	South Africa	STEU 7798	KP279299	—
*Fomitiporella* sp.	South Africa	STEU 7039	JQ038892	—
*Fomitiporella* sp.	South Africa	STEU 7038	JQ038891	—
*Fomitiporella* sp.	South Africa	STEU 7799	KP279298	—
*Fomitiporella* sp.	USA	0509/114	KX181314	KX181336
*Fomitiporella* sp.	USA	CBS 303.66	—	AY059036
*F.subinermis*	China	Dai 15114	KX181308	KX181344
*F.subinermis*	China	Dai 15131	KX181307	KX181345
*F.tenuissima*	China	Dai 12365	KC456244	KC999901
*F.tenuissima*	China	Dai 12245	KC456242	KC999902
*F.tenuissima*	China	Dai 12255	KC456243	KC999903
*F.umbrinella*	USA	JV 0312/26.6J	KX181291	—
*F.umbrinella*	USA	JV 0904/149J	KX181293	KX181329
*F.vietnamensis*	Vietnam	Dai 18377	MG657332	MG657326
*F.vietnamensis*	Vietnam	Dai 18382	MG657333	MG657327
*Inocutisdryophila*	USA	L(61)5-20-A	AM269783	AM269846
*I.dryophila*	USA	SP 25	AM269782	AM269845
